# Mechanism of the mitogenic influence of hyperinsulinemia

**DOI:** 10.1186/1758-5996-3-10

**Published:** 2011-06-13

**Authors:** Boris Draznin

**Affiliations:** 1 Division of Endocrinology, Department of medicine, University of Colorado, Denver, USA

## Abstract

Either endogenous or exogenous hyperinsulinemia in the setting of insulin resistance promotes phosphorylation and activation of farnesyltransferase, a ubiquitous enzyme that farnesylates Ras protein. Increased availability of farnesylated Ras at the plasma membrane enhances mitogenic responsiveness of cells to various growth factors, thus contributing to progression of cancer and atherosclerosis. This effect is specific to insulin, but is not related to the type of insulin used. Stimulatory effect of hyperinsulinemia on farnesyltransferase in the presence of insulin resistance represents one potential mechanism responsible for mitogenicity and atherogenicity of insulin.

## Review

Insulin is a major anabolic hormone that governs carbohydrate metabolism and contributes greatly to the metabolism of lipids and proteins. Clinically, its primary role is to promote glucose utilization and regulate hepatic glucose production. At the same time, insulin is an important, albeit mild growth factor. It promotes cell growth, cell division, migration, and inhibits apoptosis. These aspects of insulin action are collectively known as the "mitogenic actions" of insulin [1] and because they are so critical to cellular physiology, insulin is always present in cell culture medium for the propagation and maintenance of cells in culture. Although insulin is a much weaker mitogen [2] than insulin-like growth factors (IGFs), platelet-derived growth factor (PDGF), vascular endothelial growth factor (VEGF) and epidermal growth factor (EGF), insulin has a very specific mitogenic action that in fact modulates cellular responsiveness to all other growth factors, potentiating other growth factors' action [3,4].

**Figure 1:**
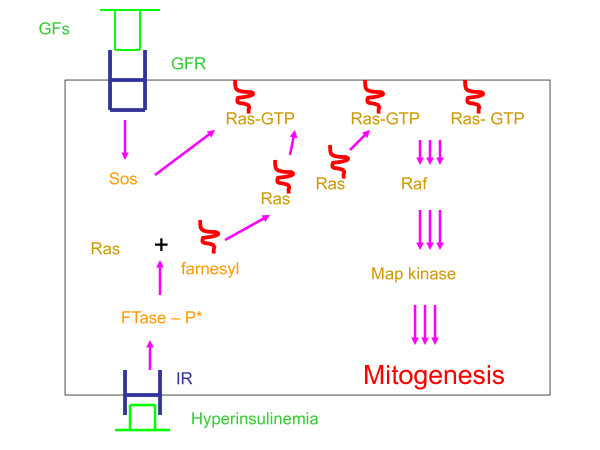
**Insulin potentiates cellular mitogenic responsiveness to growth factors (GFs)**. GFs (including insulin) interact with their cognate growth factor receptors (GFR) and activate the Sos-Ras-Raf-Map kinase branch of cellular signalling, thus promoting mitogenesis. Initially, GFs stimulate guanine nucleotide exchange factor (Sos) that activates Ras proteins by GTP loading. Sos promotes exchange of GTP for GDP only if Ras is anchored at the plasma membrane. Translocation of Ras protein to the plasma membrane is facilitated by farnesylation of Ras, an attachment of a farnesyl moiety to cysteine residue of Ras under the influence of farnesyltransferase (FTase). Insulin via interaction with its specific cell surface receptor (IR) phosphorylates and activates FTase that increases farnesylation of Ras. Hyperinsulinemia, particularly in the presence of insulin resistance, stimulates FTase and augments the amount of farnesylated Ras available for GTP loading in response to other growth factors. Greater activation of Ras leads to increased mitogenic responsiveness of cells and tissues and enhanced mitogenesis and subsequent pathophysiologic events. Reprinted with permission from Diabetologia 53:229-233, 2010.

Let us now briefly review the molecular mechanisms by which insulin and hyperinsulinemia, particularly when it occurs in the setting of insulin resistance, can augment proliferative events. In order for all growth factors to stimulate mitogenesis, they must activate the Ras-Raf-Map kinase signalling pathway (Figure 1). Ras proteins are activated by binding guanosine triphosphate (GTP), a process promoted by the guanine nucleotide exchange factor, Sos. This activation can occur only if Ras proteins are anchored at the plasma membrane (Figure 1) [5].

Isoprenylation of Ras is the first step that commits Ras to the process of translocation to the plasma membrane (Figure 1) [reviewed in [6]]. Because isoprenylation of Ras involves an attachment of a farnesyl moiety (a 15-carbon intermediary in the cholesterol synthesis pathway), the process is also known as farnesylation and is activated by the enzyme farnesyltransferase (FTase). Farnesylated Ras is then destined to anchor at the plasma membrane where it can be activated by growth factors.

As in the case of other growth factors, insulin activates Sos with a subsequent activation of farnesylated Ras and other downstream targets. However, unlike other growth factors, insulin also activates FTase by phosphorylating its alpha subunit [7,8]. Phosphorylation and activation of FTase increases the amounts of membrane-bound, farnesylated Ras that are available for activation by other growth factors (Figure 1) [9]. This effect of insulin on FTase is specific for insulin and is not mimicked by other growth factors [3]. Moreover, insulin's activation of FTase requires an intact insulin, but not IGF-1, receptor, indicating that this action is mediated exclusively by the insulin receptor and is not an ancillary effect of insulin/IGF-1 receptor interaction. This was demonstrated in cells expressing the chimeric insulin/IGF-1 receptor and in cells derived from the insulin receptor knockout animals [3]. Furthermore, in the context of insulin resistance, whereby the canonical phosphatidylinositol 3-kinase (PI3K)/Akt metabolic pathway of insulin signalling is inhibited to various degrees, the Ras/Raf/Map kinase mitogenic pathway of insulin is undisturbed and possibly upregulated, leading to increased insulin-stimulated activation of FTase with subsequent increases in the amounts of farnesylated Ras [8,9]. Taken together, these actions of insulin, although normal within the context of insulin signaling enhance the mitogenic responsiveness of cells and tissues.

The crux of the matter is that hyperinsulinemia, whether in cell culture or in vivo (i.e., in animals and humans), leads to the overstimulation of FTase and excessive farnesylation and membrane association of Ras proteins, whereby increasing cellular responsiveness to other growth factors. This potentiation of the mitogenic effects of other growth factors becomes critical in the pathophysiology of progression of cancer and vascular disease [9-11].

Several in vivo studies provided observational and experimental support to this hypothesis. Thus, liver, aorta, and skeletal muscle of the insulin resistant ob/ob mice and fa/fa rats were found to contain increased amounts of farnesylated Ras [12]. Reduction of hyperinsulinemia by exercise training resulted in decreased amounts of farnesylated Ras in Zucker fa/fa rats. Induction of fetal hyperinsulinemia by direct infusion of insulin into the fetus by either fetal or maternal infusions of glucose resulted in significant increases in the activity of FTase and the amounts of farnesylated Ras in fetal liver, skeletal muscle, fat, and white blood cells [13]. An additional infusion of somatostatin into hyperinsulinemic fetuses blocked fetal hyperinsulinemia and completely prevented these increases, specifying insulin as a causative factor. In other studies, insulin infusions significantly increased the amounts of farnesylated Ras in white blood cells of humans, in liver samples of mice and dogs, and in aorta samples of mice [4]. Taken together, these findings strongly support the in vivo relationship between insulin resistance and ability of insulin to stimulate FTase activity.

Overall, we have observed the ability of insulin to potentiate action of IGF-1, EGF, PDGF and VEGF in variety of tissues, including vascular smooth muscle cells, endothelial cells, adipocytes, fibroblasts, liver cells, and breast cancer cells [3,4,7,9-15]. This effect of insulin has been consistently observed whenever metabolic insulin resistance along the PI 3-kinase pathway is present. Thus, enhanced cellular responsiveness to growth factors is a physiologic effect of insulin that crosses over into a pathological one in response to hyperinsulinemia, whether endogenous (secondary to insulin resistance) or exogenous (secondary to chronic iatrogenic overinsulinization of insulin resistant individuals).

The question that lies before us is - What are the clinical implications of the hypothesis that hyperinsulinemia, in a setting of insulin resistance, exerts significant mitogenic and pro-atherogenic influence? Clearly, acceptance of this postulate would demand an aggressive correction of insulin resistance in order to diminish endogenous compensatory hyperinsulinemia and to minimize exogenous hyperinsulinemia. The most appropriate way of addressing insulin resistance therapeutically is to stress lifestyle modifications - diet and physical activity. Dietary compliance must return to its proper place as a cornerstone of diabetes therapy [16], while the practice of covering dietary indiscretions with increasing doses of insulin should be discouraged.

Most patients with Type 2 diabetes are insulin resistant, and "paying lip service" to dietary and lifestyle therapies leads to overinsulinization. Many patients with Type 1 diabetes who use large doses of insulin to cover for their excessive intake of carbohydrates are also insulin resistant. Carbohydrate counting is an extremely useful tool in diabetes therapy, but consuming dietary carbohydrate without limits also leads to overinsulinization. Use of insulin sensitizers, such as Metformin, would be the second best approach to improving insulin resistance.

Several studies have demonstrated the increased likelihood of developing cancer in patients treated with insulin or insulin secretagogues as compared with Metformin [17,18]. The data collected between 1991 and 1996 demonstrated that patients with type 2 diabetes mellitus exposed to sulfonylureas and exogenous insulin had significantly greater cancer-related mortality than did patients treated with metformin [18]. Recently, Jiralespong and colleagues [19] have compared the rates of pathologic complete response (pCR) in diabetic patients with breast cancer receiving neoadjuvant chemotherapy and treated with metformin with those not treated with metformin. The rate of pCR (better outcomes) was 24% in the metformin group and 8% in the non-metformin group (p = 0.007). While the use of insulin did not influence the rate of pCR in the metformin-treated group, its use in the non-metformin group was associated with the lowest rate of pCR (p = 0.05).

Undoubtedly, insulin (human, pork, beef, and analogues) per se does not cause cancer or atherosclerosis. If anything, insulin, a life-saving hormone, has improved dramatically the life expectancy of patients with diabetes. However, high physiological concentrations of insulin can substantially increase cellular mitogenic responsiveness to other growth factors and promote disadvantageous growth and proliferation, particularly in the presence of insulin resistance.

The constellation of the metabolic insulin resistance (diminished strength of insulin signaling along the PI 3-kinase branch of its action) and hyperinsulinemia results in overstimulation of the MAP kinase signaling branch and chronic activation of FTase with a subsequent increases in the amounts of farnesylated Ras. These events augment the mitogenicity of other growth factors, thereby promoting the progression of cancer and atherosclerosis [20].

In summary, the detrimental mitogenic effects of hyperinsulinemia must be addressed along with hyperglycaemia when treating diabetes. Endogenous hyperinsulinemia must be treated by minimizing insulin resistance with diet, exercise and insulin sensitizing medications, whereas exogenous hyperinsulinemia must be avoided by selecting appropriate diet and life style while using minimal doses of insulin to achieve normoglycaemia. Inducing hyperinsulinemia as a price for paying "lip service" to dietary therapy is not only inexcusable, but also potentially harmful.

## Competing interests

The author declares that they have no competing interests.
